# Fourier-transform infrared spectroscopy for rapid *Streptococcus pneumoniae* serotyping in a tertiary care general hospital

**DOI:** 10.3389/fmicb.2025.1565888

**Published:** 2025-04-23

**Authors:** Miriam Campos-Ruiz, Jun Hao Wang-Wang, Antoni E. Bordoy, Beatriz Rodríguez-Ponga, Natalia Pagan, Jessica Hidalgo, María Dolores Quesada, Montserrat Giménez, Pere-Joan Cardona

**Affiliations:** ^1^Microbiology Department, Laboratori Clínic Metropolitana Nord, Germans Trias i Pujol University Hospital, Badalona, Spain; ^2^Genetics and Microbiology Department, Universitat Autònoma de Barcelona, Bellaterra, Spain; ^3^Germans Trias i Pujol Research Institute (IGTP), Badalona, Spain; ^4^Centro de Investigación Biomédica en Red en Epidemiología y Salud Pública (CIBERESP), Instituto de Salud Carlos III (ISCIII), Madrid, Spain; ^5^Centro de Investigación Biomédica en Red en Enfermedades Respiratorias (CIBERES), Instituto de Salud Carlos III (ISCIII), Madrid, Spain

**Keywords:** Fourier-transform infrared spectroscopy, FTIR, *Streptococcus pneumoniae*, Quellung, artificial intelligence, serotyping

## Abstract

*Streptococcus pneumoniae* is the leading cause of community-acquired pneumonia and remains a significant contributor to bacteremia and meningitis, collectively known as invasive pneumococcal disease (IPD). Certain serotypes are more strongly associated with severe illness and antimicrobial resistance. Accurate serotyping is essential for effective IPD surveillance and vaccine development. Fourier-transform infrared (FTIR) spectroscopy has emerged as a valuable tool for differentiating among serotypes across various isolates. We analyzed 150 pneumococcal strains isolated from a tertiary hospital in Barcelona, Catalonia, Spain, between 2016 and 2023, representing 32 serotypes associated with IPD. Forty-nine samples (33%) exhibited serotypes included in PCV13 vaccine. Each strain was classified using (A) FTIR-based clustering and (B) FTIR machine-learning-based PneumoClassifier algorithm. The results were compared to the Quellung reaction, the gold standard methodology. Clustering method grouped correctly PCV13-serotypes 1, 3, and 19F and non-PCV13 serotypes 6C, 7BC, 17F, 24F, 31, and 35B (48/150). PneumoClasifier algorithm successfully grouped all PCV13-serotypes (49/49) including some of the most virulent described serotypes, such as 1, 6B, 7F, and 14. Among non-PCV13 serotypes, it correctly classified 73 out of 101 isolates (72.3%). However, 12F, 15AB, 16F, 17F, 23A, and 24F were misclassified. Overall, PneumoClassifier achieved an accuracy of 122/150 (79.80%) in serotyping pneumococcal strains, demonstrating higher concordance with Quellung (adjusted Rand index: 0.717, adjusted Wallace coefficient: 0.636) compared to the clustering approach (0.397 and 0.378, respectively) (*p* < 0.001). FTIR has proven to be a rapid, user-friendly, cost-effective, and practical technique, making it a promising first-line tool for *S. pneumoniae* serotyping.

## 1 Introduction

*Streptococcus pneumoniae* is an opportunistic pathogen responsible for at least half of all community-acquired pneumonia and otitis media and remains a significant cause of invasive pneumococcal disease (IPD) resulting in around one million deaths annually that affect mainly children and older people (Janoff and Musher, 2015; Liu et al., 2016; Weiser et al., 2018).

The capsule of *S. pneumoniae* is composed of high-molecular-weight polysaccharide polymers that are unique and distinctly antigenic for each of more than 100 serotypes of *S. pneumoniae* strains (Geno et al., 2020). Nevertheless, only a limited number of pneumococcal serotypes cause the majority of IPD around the world. In Europe, the 10 most common serotypes involved in IPD were 3, 6C, 8, 9N, 10A, 11A, 12F, 15A, 19A, 22F, and 23B, accounting for 70% of all cases of IPD with a known serotype in 2018 (European Centre for Disease Prevention and Control, 2020). Furthermore, some studies have shown that specific serotypes, such as 1, 6B, 7F, 14, and 19 are more virulent and more strongly associated with invasiveness and complications such as meningitis (Brueggemann et al., 2003; Pérez et al., 2004; Sandgren et al., 2004). Some circulating pneumococcal serotypes are also more related to resistance to antibiotics (Yahiaoui et al., 2018).

Knowing the circulating serotypes is important for understanding the local epidemiology and developing vaccines that target dominant *S. pneumoniae* strains in IPD. In Catalonia since 1999 the PSV23 vaccine has been recommended for people at risk of acquiring IPD and people above the age of 65. The PCV13 vaccine was incorporated into the Catalan systematic vaccination program in 2016 for children under 2 years old, leading to a vaccination coverage of 81.8% in 2017 and 91% in 2020 (Subdirecció General de Vigilància i Resposta a Emergències de Salut Pública, 2022). Interestingly, serotype 3, which is included in PCV13 and isolated from IPD cases in both adults and children, remained stable in Catalonia for over three decades despite the introduction of PCV13 in the pediatric population (Subdirecció General de Vigilància i Resposta a Emergències de Salut Pública, 2022; Calvo-Silveria et al., 2024). Since 2023, PCV15 has replaced PCV13 in the vaccination program for the pediatric population, while PCV20 has been approved for individuals over 65 years old and those with risk factors for IPD.

The gold standard method for *S. pneumoniae* serotyping is the Quellung reaction (SS Diagnostica, Denmark) (Habib et al., 2014). This phenotypic technique involves the use of polyclonal antisera and the visualization of the reaction using light microscopy to classify the pneumococcal strains into specific serotypes. This method is expensive, requires well-trained technicians, labor intensive and they can involve subjective interpretation. That’s why it is unsuitable for high throughput serotyping and their use is restricted to reference centers. To solve all these inconveniences, new techniques based on molecular biology have been developed.

Capsulation locus (cps) determines the serotypes of *S. pneumoniae* by amplifying and sequencing specific regions of the bacterial DNA, followed by comparing these sequences with a reference database to assign a serotype. Serotype assignment accuracy relies on a precise reference database and can be challenging for serotypes with highly conserved *cpsB* regions, potentially reducing resolution in some cases (Leung et al., 2012). Multiplex real-time PCR are more sensitive than *cpsB* typing being able to identify up to 34 serotypes (Velusamy et al., 2020). Finally, whole-genome sequencing achieves a sensitivity of 86–100% but is an expensive and it can have long turnaround times in the daily routine of a clinical microbiology laboratory (Knight et al., 2021).

Fourier transform infrared (FTIR) spectroscopy has been used to distinguish encapsulated from unencapsulated strains of *S. pneumoniae* (Sahu et al., 2006) and recently some efforts have been done to evaluate *S. pneumoniae* serotyping (Vaz et al., 2013; Burckhardt et al., 2019; Vasconcelos et al., 2024). FTIR spectroscopy is a phenotypic technique that uses infrared light to target the sample and generate an infrared spectrum based on the absorption of infrared light. Each spectrum acts as a bacterial fingerprint and varies depending on the composition of the functional groups in the sample, such as carbohydrates, lipids, and proteins (Naumann et al., 1991; Griffiths and de Haseth, 2006). The region between wave numbers 800 and 1,300 cm^−1^, where the carbohydrates of the whole cell predominantly absorb, enables capsular typing (Burckhardt et al., 2019; Passaris et al., 2022). FTIR spectroscopy is used by the IR Biotyper system (Bruker GmbH, Leipzig, Germany), which offers a rapid and low-cost typing technique (both sample processing and number of reagents are minimal) with a short turn-around time (3–4 h).

The aim of the present study was to evaluate the performance of the IR Biotyper as a rapid serotyping tool compared with the Quellung technique as the gold standard method.

## 2 Materials and methods

### 2.1 Study design

A retrospective study was performed in a tertiary care hospital. This center is the reference hospital for 1,200,000 inhabitants of the Northern Metropolitan Area of Barcelona (Catalonia, Spain).

A representative sample of 150 pneumococcal strains covering 32 serotypes were included in the present study. A minimum of three isolates for each serotype, or if not possible, at least three isolates per serogroup were included in the study set (Table 1). All *S. pneumoniae* strains were obtained from invasive samples (blood culture, cerebrospinal fluid and pleural liquid) of patients with IPD admitted to the hospital from 2016 to 2023.

**Table 1:** Serotypes represented in the tested cohort and the respective numbers of strains used.

Serotype (*N* = 150)	No. of strains in evaluated set
1	4
3	17
4	3
6A	1
6B	2
6C	3
7B	4
7C	2
7F	3
8	18
9N	5
9V	2
10A	4
11A	5
12F	3
14	4
15A	4
15B	3
16F	5
17F	3
19A	5
19F	5
22F	8
23A	5
23B	4
23F	3
24F	3
31	5
33F	5
35B	2
35F	3
38	7

**Figure 1:**
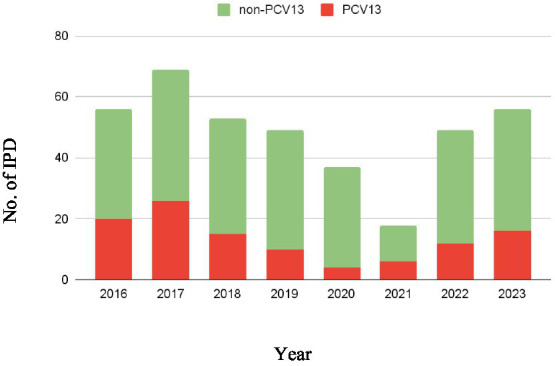
Isolated serotypes in IPD during the study period (2016–2023). The height of each column represents the total number of IPD isolates for the corresponding year. Non-PCV13 serotypes are shown in green, while PCV13-included serotypes are shown in red.

During the study period (2016–2023) 389 *S. pneumoniae* strains were isolated from IPD, mainly with a respiratory focus. The total number of cases of IPD at our hospital increased from 2016 to 2017, reaching the highest IPD case number during the entire study period. Subsequently, the number of cases decreased, with a slight uptick observed in 2022 and 2023 (Figure 1). During the study period the pneumococcal serotypes included in the PCV13 vaccine were less frequently isolated in IPD than those not included in this vaccine (Figure 1). The most frequently isolated serotypes were the 8 (14.91%), which is not included in PCV13, and the 3 (11.57%), which is included.

This study was approved by the Clinical Research Ethics Committee (CEIC) of the University Hospital Germans Trias i Pujol in Badalona, Spain (PI-24-287).

In the present study, informed consent was waived since no additional samples were required for the evaluation, no clinical report was modified, and no patient data was used.

### 2.2 Routine microbiological diagnostic

All isolates were initially identified at the species level by matrix-assisted laser desorption ionization-time of flight mass spectrometry (MALDI-TOF MS, Bruker Daltonik GmbH, Bremen, Germany). Identification of *S. pneumoniae* was confirmed in parallel using the Optochin Susceptibility Test (Bio-Rad Laboratories, Inc., California, United States).

All *S. pneumoniae* strains are routinely sent to the National Centre of Microbiology (CNM) [Institute of Health Carlos III (ISCIII), Madrid, Spain] for the Quellung reaction (gold standard method).

All *S. pneumoniae* strains are routinely preserved in a homemade storage media based on commercial milk at −80°C until further analysis. The homemade medium is prepared using commercial Infasource^®^ milk (Ref. 505023; Nestlé S.A., Vevey, Switzerland). Specifically, 1 mL of milk is distributed into autoclavable glass tubes and sterilized in an autoclave at 121°C for 20 min. Subsequently, the tubes are stored in a refrigerator at 6°C ± 2°C.

### 2.3 Sample preparation for FTIR analysis

All *S. pneumoniae* isolates were defrosted and cultured in Columbia CNA agar medium (bioMérieux, Marcy-l’Étoile, France) for 24 h at 37°C in 5% CO_2_. The next day, isolates were subcultured on fresh sheep blood agar culture (bioMérieux, Marcy-l’Étoile, France) in the same conditions.

Pneumococcal strains were prepared for FTIR spectroscopy using the direct smear technique according to Passaris et al. (2022) methodology. Briefly, 1-μL plastic loop was used to carefully remove the biomass from the plate and apply it to in triplicate on a 96-spot silicon plate (BrukerDaltonics GmbH, Bremen, Germany). As quality control, 12 μL of two *Escherichia coli* strains (IRTS1 and IRTS2; IR Biotyper^®^ kit, Bruker) were placed in duplicate on the same silicon plate to validate the run. The plate was dried for 15 min at 37°C and placed under a UV lamp for 10 min to inactivate the bacterial cells. Finally, the plate was inserted into the IR Biotyper (Bruker, Germany).

### 2.4 Spectrum analysis of FTIR data

All samples were tested in triplicate using the default analysis setting. Spectra were acquired in transmission mode (wavelength region 500–4.000 cm^−1^) using OPUS 8.2.28 software (Bruker GmbH, Leipzig, Germany). For spectra analysis, the default splicing method using the polysaccharide region (wavelength region 1,300–1,800 cm^−1^) was applied.

The spectrum analysis was performed following two different approaches:

- FTIR clustering method: Firstly, the average of all spectra of each individual strain was performed using the OPUS software. The mean spectra were used to create a dendrogram based on hierarchical cluster analysis using the Euclidean distance metric and the average linkage method (UPGMA) without dimensionality reduction techniques. For *S. pneumoniae*, the manufacturer suggests a cut-off value ranging from 0.20 to 0.25 for clustering. A validation to determine the best cut-off was performed using Quellung technique. A subset of the first isolates analyzed by the FTIR (*n* = 65) was used to determine the best cut-off value that maximized the overall congruence between FTIR and Quellung results. To calculate the best cut-off that grouped strains with the same serotype and separated those with different serotypes, the adjusted Rand index (AR) was calculated for the comparison between the Quellung technique and the clustering system for each selected cut-off between 0.15 and 0.225 (Supplementary Figure 1). When two or more isolates had a FTIR spectrum distance less or equal to the designated cut-off value, they were considered to belong to the same FTIR cluster. Conversely, they were designated FTIR singletons.
  Subsequently, the analysis was performed using dimensionality reduction methods. Specifically, the following analyses were conducted: (A) principal component analysis (PCA) with 40 components explaining 99% of the variability. (B) Linear discriminant analysis (LDA) with 40 components explaining 99% of the variability. Each isolate was assigned its serotype result obtained through the Quellung reaction as a class label. For each type of analysis, an optimal cut-off study was performed in the same manner as previously explained (Supplementary Figures 2, 3). A dendrogram was then constructed based on hierarchical cluster analysis using the Euclidean distance metric and the average linkage method (UPGMA), incorporating the corresponding dimensionality reduction technique.

- Artificial intelligence model (PneumoClassifier, version 4.0): The FTIR offers the machine-learning based algorithms to classify the spectra of each isolate in the different serotypes. Furthermore, the algorithm generates a score (ranging from 0 to 100) that assesses the reliability of the serotype being displayed. The version used was trained with 95 strains from 34 different serotypes: 1, 3, 4, 5, 6ABCD, 7F, 8, 9N, 9V, 10A, 11A, 12F, 14, 15ABC, 16F, 17F, 19AF, 20, 22F, 23ABF, 24F, 31, 33F, and 35BF. The PneumoClassifier incorporates an additional AI algorithm for subtyping the serotypes within serogroup 6 into 6A, 6B, and 6CD. However, it cannot subtype serotypes within serogroups 15, 19, 23, and 35.
  The pneumococcal strains were considered correctly classified if the AI algorithm identified them with the same serogroup and/or serotype as determined by the Quellung reaction.

### 2.5 Concordance between FTIR and gold standard results

To assess the concordance of the clustering and PneumoClassifier results obtained with FTIR and the Quellung serotyping, the adjusted Rand index (AR) and the adjusted Wallace coefficients (AW) were calculated with 95% CI using an online tool (Comparing Partitions, 2009; available at: http://www.comparingpartitions.info/?link=Tool). AR compares the overall congruence between two compared methods (Santos and Embrechts, 2009), while AW compares the agreement of the two approaches considering one of them as the reference method (Severiano et al., 2011). AR and AW values may range from 0 to 1, where 0 means agreement expected by chance and 1 means perfect correlation between both methods (Severiano et al., 2011). Additionally, *p*-values comparing the AW and AR values for (I) FTIR clustering approach vs. Quellung and (II) PneumoClassifier vs. Quellung were obtained according to the jack-knife pseudo-values resampling method (Comparing Partitions, 2009).

## 3 Results

### 3.1 Spectrum analysis using FTIR clustering method

**Figure 2:**
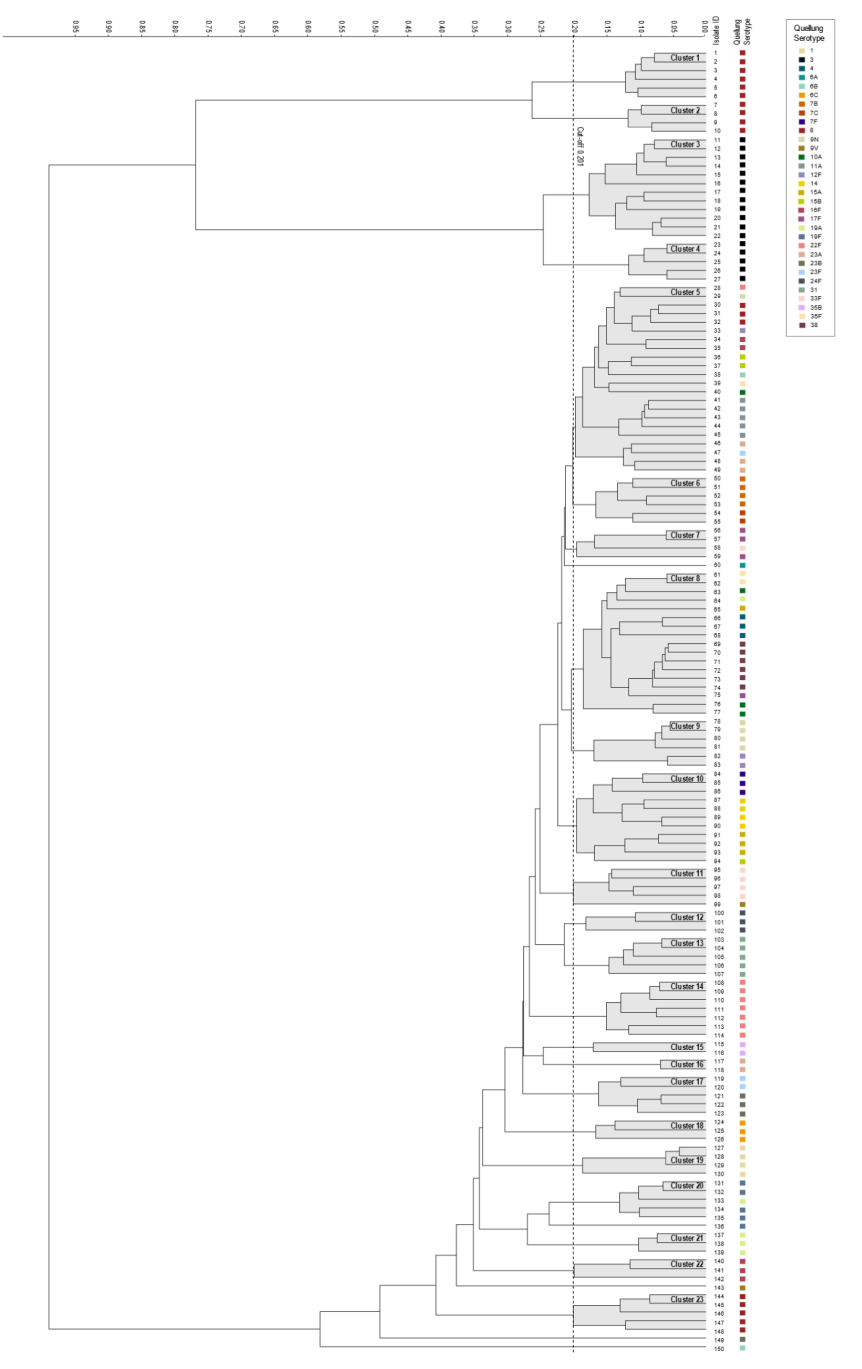
Dendrogram visualizing the 150 pneumococcal tested strains representing 32 serotypes. The vertical dashed line depicts the cutoff value (0.201). The linkage type used for the dendrogram was Euclidean distance. Resulting FTIR clusters (1–23) are shadowed in gray. Corresponding serotype are indicated in each isolate.

For FTIR clustering, an optimal cut-off of 0.201 was obtained after an initial validation (*n* = 65) (Supplementary Figure 1). The 150 pneumococcal strains were classified in 23 clusters and 5 singletons (Figure 2). Three of the largest cluster were cluster 5 (*n* = 22), 8 (*n* = 17), and 10 (*n* = 11); each of them include pneumococcal strains with serotypes 4, 6B, 7F, 8, 9N, 10A, 11A, 12F, 14, 15 (A, B), 16F, 17F, 19A, 22F 23 (A, F), 35F, and 38, suggesting low discrimination between these pneumococcal serotypes. Conversely, cluster 3 included 12 out of 17 pneumococcal strains with PCV13-serotype 3 and were closely related with cluster 4 which included 5 out of 17 strains of serotype 3 (Figure 2). Other serotypes grouped properly were the PCV13-included serotype 1 (cluster 19) and non-PCV13 serotypes 6C (cluster 18), 7BC (cluster 6), 17F (cluster 7), 24F (cluster 12), 31 (cluster 13) and 35B (cluster 15) (Figure 2). All of this translates to 43 out of 150 pneumococcal strains being correctly classified, with AR and AW values of 0.397 (95% CI, 0.280–0.511) and 0.378 (95% CI, 0.217–0.523), respectively.

When dimensionality reduction techniques were applied, the following results were obtained: (A) For PCA, AR and AW values of 0.469 (95% CI, 0.368–0.566) and 0.347 (95% CI, 0.297–0.398), respectively, were obtained, without achieving statistical significance compared to clustering performed without dimensionality reduction techniques (*p* = 0.202) (Supplementary Figure 4). (B) For LDA, AR, and AW values of 0.950 (95% CI, 0.908–0.994) and 0.905 (95% CI, 0.877–0.933), respectively, were obtained, significantly improving clustering performance (*p* < 0.001) (Supplementary Figure 5).

### 3.2 Pneumococcal serotyping using PneumoClassifier approach

**Figure 3:**
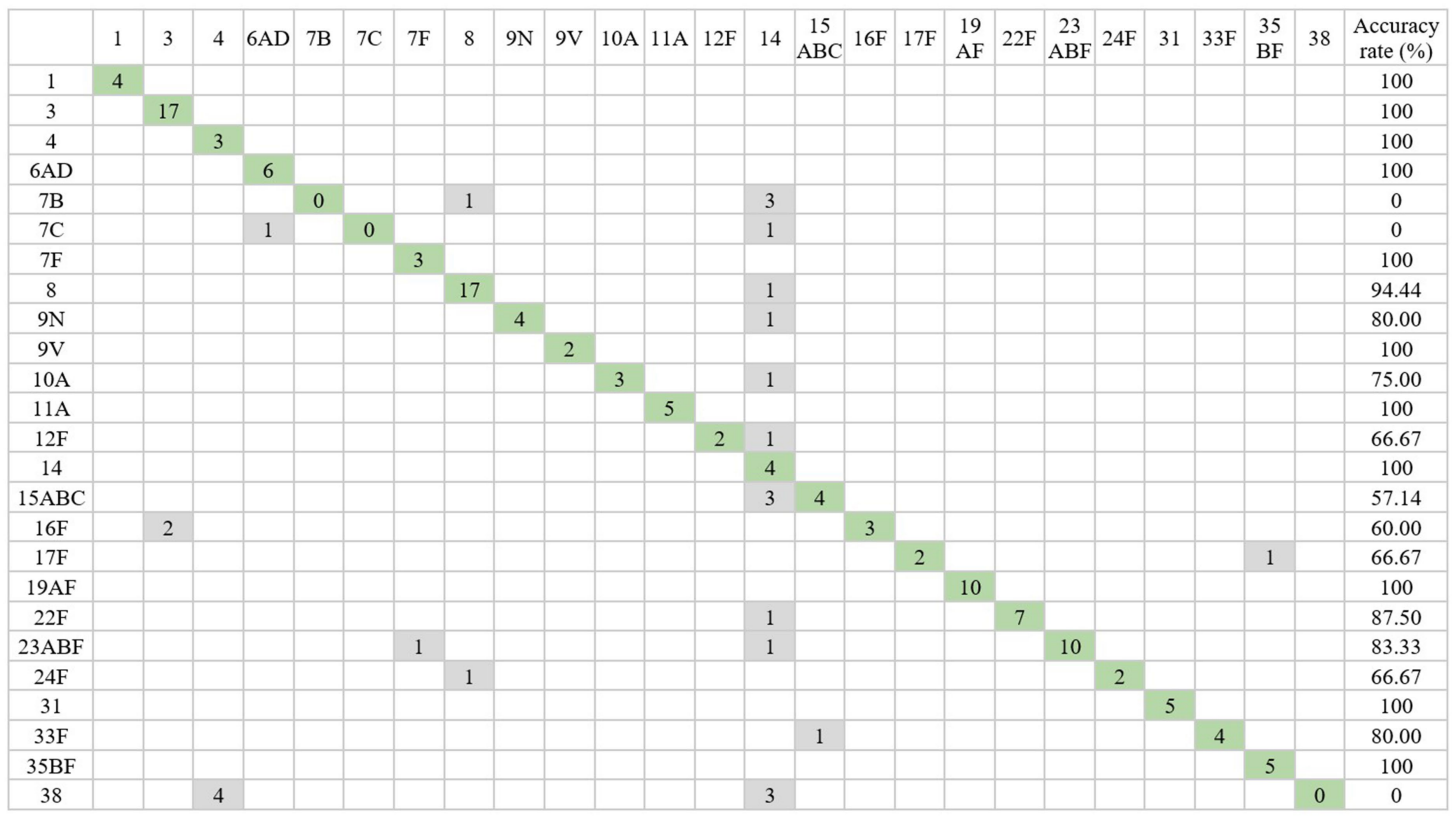
Confusion matrix with the serotyping results obtained from PneumoClassifier version 4.0 compared with those obtained with Quellung reaction. The cells highlighted in green represent the strains correctly identified by the PneumoClassifier. The isolates incorrectly typed are shown in gray.

For the FTIR PneumoClasifier, it correctly predicted the serotype of 122 out of 150 (79.80%) pneumococcal strains. All isolates belonging to serotypes 1, 3, 4, 6ABC, 7F, 9V, 11A, 14, 19AF, 23BF, 31, and 35BF were correctly classified (Figure 3). Our study set includes six strains belonging to serogroup 6: 6A (*n* = 1), 6B (*n* = 2), and 6C (*n* = 3). The additional PneumoClassifier algorithm successfully subtyped 5 out of 6 strains, with one 6B strain remaining misclassified. However, this strain was correctly classified within the 6AD group.

Serotypes 8 and 22F were correctly classified in 94.44 and 87.50% of cases, respectively, and only one pneumococcal strain of each serotype was classified as serotype 14 (Figure 3 and Table 2). Serotypes 9N, 10A, 12F, 15AB, 16F, 17F, 23A, 24F, and 33F were correctly classified in 50–80% of the cases (Figure 3).

**Table 2:** Summary of the 15 *S. pneumoniae* isolates that were mistyped by the PneumoClassifier.

Serotype from Quellung reaction	No. of strains	No. of mistyped strains (*n* = 15)	Serotype from FTIR (No. of strains)
8	18	1	14 (1)
9N	5	1	14 (1)
10A	4	1	14 (1)
12F	3	1	14 (1)
15A	4	2	14 (2)
15B	3	1	14 (1)
16F	5	2	3 (2)
17F	3	1	35BF (1)
22F	8	1	14 (1)
23A	5	2	14 (1), 7F (1)
24F	3	1	8 (1)
33F	5	1	15 ABC (1)

Regarding the 28 discordant results, 13 samples belonged to serotype 7 (B, C) and 38 could not be classified by the AI system because these serotypes had not been included in the PneumoClasifier training set (Figure 3). Conversely, the 15 remaining discordant results belonged to serotypes that are included in the PneumoClassifier training set. Among them, they were frequently mistyped as serotype 14 (9/15). The other confounded serotypes were serotype 3 (2/15), 7F (1/15), 8 (1/15), 15 (1/15) and 35 (1/15). The detailed information of each mistyped sample is listed in Table 2.

Overall, the PneumoClassifier accurately classified all pneumococcal strains with serotypes included in the PCV13 (49/49; 100%) and 73 out of 101 strains (72.3%) with serotypes not included in the PCV13 vaccine.

The AR and AW values comparing the PneumoClassifier results with the Quellung technique were 0.717 (95% CI, 0.580–0.855) and 0.636 (95% CI, 0.464–0.803), respectively. When strains with serotype 7BC and 38 were excluded from the analysis, these values slightly increased to 0.806 (95% CI, 0.693–0.923) for AR and 0.770 (95% CI, 0.624–0.915) for AW. Overall, the PneumoClassifier demonstrated significantly greater concordance with the Quellung technique compared to the clustering system (*p* < 0.001), whether or not the 7BC and 38 serotypes were excluded.

## 4 Discussion

This study demonstrates the potential of FTIR spectroscopy as a tool for serotyping *S. pneumoniae*, particularly while awaiting results from the Quellung reaction. Among the 150 pneumococcal strains analysed, the PneumoClassifier correctly identified 122 strains to the serotype level, achieving and accuracy of 79.80%, consistent with previous studies (Burckhardt et al., 2019; Passaris et al., 2022; Vasconcelos et al., 2024). When comparing the performance of FTIR in our study with previous studies, we obtained a similar level of concordance to Burckhardt et al. (2019) study (79.80% vs. 77.40–89.20%) and slightly lower concordance compared to Passaris et al. (2022) and Vasconcelos et al. (2024) studies (79.80% vs. 71.20–98.00%). In these studies, the authors trained their own AI model using a dedicated dataset. It is likely that the variability between the strains used for training and validation was lower in their studies than in ours, as we used the default AI model provided by Bruker. We believe that this model may include strains that differ from those circulating in our healthcare setting, which could explain some of the observed discrepancies and a slightly lower concordance in our study compared with previous studies.

FTIR has been employed to discriminate between various bacterial serotypes or serogroups including *S. pneumoniae*, *Enterococcus faecium*, *Listeria monocytogenes*, *Salmonella enterica* and three *Legionella pneumophila* serogroups (Quintelas et al., 2018). In this study, substantial agreement between the PneumoClassifier and the Quellung method was obtained with an AR of 0.806 and an AW of 0.770 (excluding serotypes 7BC and 38). These results align with prior research on gram-positive bacteria, particularly with *E. faecium* (Teng et al., 2022; Scheier et al., 2023; Park and Ryoo, 2024) where AR values exceeded 0.54 and AW values approximated 0.95 when comparing FTIR to standard methods. Teng et al. (2022) recommend an AR of at least 0.7 for FTIR assay reproducibility. Studies on gram-negative bacteria, such as *Klebsiella pneumoniae*, have reported AR values ranging from 0.743 to 1.0 and AW values from 0.708 to 1.0 (Dinkelacker et al., 2018; Martak et al., 2019; Rakovitsky et al., 2020; Hu et al., 2021).

Collectively, these findings suggest that the PneumoClassifier demonstrates substantial agreement with the reference method and could serve as a reliable serotyping tool. However, the FTIR clustering system displayed poor agreement with Quellung-derived results, indicating a weaker performance compared to the PneumoClassifier for discriminating against pneumococcal strains. Clustering is more exploratory, detecting underlying patterns without considering labels, and it can identify subtle phenotypic variations within a specific serotype, potentially dividing it into multiple subgroups based on spectral similarity. In contrast, the AI model focuses on supervised classifications based on the data provided during training. Consequently, adding more strains from less frequently isolated serotypes could potentially improve the performance of the clustering method and enhance the discrimination between different clusters. Another way to improve the performance of the clustering system could be by applying dimensionality reduction techniques such as principal component analysis (PCA) and linear discriminant analysis (LDA). When applying PCA to our cohort, we observed no statistically significant improvement compared to HCA without dimensionality reduction techniques (*p* = 0.202). In contrast, applying LDA significantly improved the AR and AW values (*p* < 0.001). However, it is inherent to a supervised technique like LDA to increase concordance values since LDA maximizes class separation with the help of class labels (Quellung results). This clustering system using LDA can be useful when there is a large number of strains with known serotypes, allowing an unknown serotype strain to be grouped with high probability alongside other strains of the same serotype, potentially even better than HCA in our cohort. However, for real prospective use, an LDA model must first be trained using a gold-standard technique such as Quellung to retrospectively assign class labels and build a robust model with the most comprehensive representation of pneumococcal serotypes. Additionally, as with any clustering system, whether or not dimensionality reduction techniques are applied, strains with previously unanalyzed serotypes will remain unclustered (singletons) since they do not group with any other strain. In such cases, PneumoClassifier would be the only system capable of providing a result. It is important to consider that pneumococcal serotypes vary each year, as they depend on vaccine coverage. As a result, new serotypes may emerge annually that the clustering system has not yet identified.

Some discrepancies between FTIR results and the Quellung method have been previously documented by Passaris et al. (2022). Both techniques assess the capsule structure, but their underlying mechanisms differ significantly. The Quellung reaction relies on the 3-D specificity of polyclonal antibodies binding to the pneumococcal capsule, whereas FTIR spectroscopy analyses the vibrations and rotations of covalent bonds, particularly C-O stretching and O-H bending. Then, as hypothesized in previous studies, certain serotypic differences may be more easily identified by Quellung and other differences may be better detected with FTIR (Burckhardt et al., 2019; Passaris et al., 2022).

Moreover, FTIR spectroscopy measures chemical bonds not only on the pneumococcal outer surface but also within the cell, potentially making it more sensitive to detect subtle differences in glycoproteins and/or glycolipids between related strains. This added sensitivity could explain some of the variations observed. Additionally, significant genetic variation has been reported for specific serotypes (Jefferies et al., 2004; Silva et al., 2006; Lo et al., 2019). These genetic differences might manifest as minor variations among strains of the same serotype, thereby complicating clustering and reducing agreement between methods.

In this study, FTIR spectroscopy was validated for rapid serotyping in a clinical microbiology laboratory using 150 representative pneumococcal strains, marking a novel approach within our country. In Burckhardt et al. (2019) and Passaris et al. (2022) studies, they have validated the FTIR as a rapid, cost-effective, and medium-throughput alternative to the classical phenotypic techniques at research level using in house strains collection or pneumococcal strains from reference centers. The strains used in our study were obtained from invasive samples from hospitalized patients with invasive pneumococcal disease. Vasconcelos et al. (2024) use clinical strains as in our study. However, we used a higher number of strains (150 vs. 76 samples) and included a greater variety of different serotypes (34 vs. 18).

Notably, pneumococcal serotype distribution varies geographically due to factors such as antibiotic pressure and vaccination policies. Accurate and rapid serotyping of the pneumococcal isolates is crucial for several reasons. First, it enables the identification of the most frequent circulating strains in a specific region of interest, facilitating reporting to public health authorities and guiding vaccination strategies to optimize coverage. Second, it aids in the rapid identification of strains with known higher probability of resistance to beta-lactams, offering additional advantage over the gold standard method. IPD follows a seasonal pattern (with higher incidence typically in the winter months). FTIR enables fast serotyping and the optimization of empirical treatments within the same epidemic period, whereas with Quellung, logistical factors such as the need for sample transport to reference centers increase the overall response time to 2–3 months, often meaning that the epidemic period has already passed. Overall, in contrast to the Quellung method, FTIR-based serotyping enables real-time epidemiological surveillance with rapid turnaround times, optimizing local treatment guidelines for IPD.

In our cohort, the predominant pneumococcal serotypes isolated during the study period in IPD cases were non-PCV13 serotype 8 and PCV13-included serotype 3. Both were accurately classified using the PneumoClassifier and/or the dendrogram method, highlighting the reliability of FTIR-based serotyping.

In Catalonia the serotypes 14, 19F, and 6B were the main cause of IPD with beta lactam resistance until the appearance of PCV7 vaccine. Then, these serotypes were replaced by 19A associated with resistance to beta lactams and macrolides. After the introduction of PCV13 vaccine the 11A and 24F are related to amoxicillin and cefotaxime resistance (Programas de Vigilancia Microbiológica Centro Nacional de Microbiología, 2023). FTIR was able to properly classify all these more resistant serotypes. Additionally, those serogroups/serotypes associated with the highest invasiveness and virulence such as 1, 6, 7F, 14, and 19 were correctly classified using FTIR.

The Quellung technique is a laborious approach that requires experienced personnel for the interpretation of the results, which can sometimes be subjective. Conversely, FTIR is a cost-effective strategy (approximately 17€ for FTIR vs. 60€ for Quellung per sample) that does not require highly experienced technical personnel, although an initial financial investment is required; the results obtained from the pneumococcal colonies are objective and can be obtained in less than an hour. Regarding the processing time, Quellung and FTIR are similar, but Quellung’s main limitation is its long turnaround time, which may be due to logistical factors such as the need for sample transport to reference centers, thus increasing both time and cost. While this issue may not be universal, in our facility, Quellung results from a positive culture can take months, whereas FTIR provides results within hours. This makes the FTIR-Biotyper a rapid serotyping method with significant potential for application in hospitals and public health strategies.

Some limitations of the present study include its single-center design, and the use of strains obtained from retrospectively frozen isolates, which rendered some pneumococci nonviable in culture. The PneumoClassifier requires strain growth on agar plates, in contrast to sequencing methods. Nevertheless, the majority of IPD diagnoses are still performed using cultures (Subdirecció General de Vigilància i Resposta a Emergències de Salut Pública, 2022). Furthermore, the serotypes used were the most common in our region and obtaining other serotypes from other geographical regions was not possible in this work; so, a limited number of strains for certain serotypes were included, which may have contributed to the low concordance between the clustering method and the Quellung technique, as well as to some discrepancies between PneumoClassifier and Quellung results. Variations in genetic factors, as well as the local epidemiology of pneumococcal infections, might contribute to differences in the FTIR spectral profiles of these isolates even with the same serotype. Then, these results cannot be extrapolated to other geographical regions that may have other serotypes due to different epidemiological features and vaccination policies related to *S. pneumoniae*. Therefore, each area should perform a previous validation of the PneumoClassifier with more prevalent *S. pneumoniae* strains because the training set included in the AI algorithm can be insufficient. Finally, there is a possibility that the reported discrepancies in our study may be due to erroneous Quellung results that could not be verified using another technique, like whole-genome sequencing.

Considering all these limitations, the next step to improve FTIR performance could be to develop our own AI model, for example, using LDA methodologies, incorporating a larger number of circulating strains from our region. Also, future studies involving isolates from multiple regions would be interesting to explore whether FTIR performance varies across geographical areas within the same pneumococcal serotypes.

## 5 Conclusion

FTIR spectroscopy presents a potentially user-friendly and cost-effective tool for serotyping *S. pneumoniae* in clinical microbiology laboratories, making it a viable first-line approach for the rapid identification of isolates while awaiting confirmation via the Quellung reaction. Its added value over the Quellung method lies in its ability to rapidly identify the most prevalent circulating strains, those with greater beta-lactam resistance and strains associated with higher invasiveness.

Before implementing FTIR for *S. pneumoniae* serotyping, however, it is crucial to validate the technique using the most frequent local serotypes. These serotypes may vary due to local epidemiology factors, including vaccination policies, antibiotic usage patterns, and population-specific risk factors.

## Data Availability

The original contributions presented in the study are included in the article/Supplementary material, further inquiries can be directed to the corresponding author.
