# Supplementary material for: Fourier-transform infrared spectroscopy for rapid Streptococcus pneumoniae serotyping in a tertiary care general hospital
Source: Front Microbiol. 2025 Apr 23;16:1565888. doi: 10.3389/fmicb.2025.1565888 (PMC12055794; doi:10.3389/fmicb.2025.1565888)
Supplement: Supplementary file 1 [file Data_Sheet_1.docx]

Supplementary Material

# Supplementary Figures

AR

Cut-off

**Supplementary Figure 1.** Final internal validation of FTIR clustering cut-off value. The most restrictive cut-off value that maximized the adjusted rand index (AR) was 0.201 for the clustering of the study dataset (*n*=65) of *S. pneumoniae* isolates considering Quellung reaction as the reference method. The tested cut-off range corresponds to the unofficial range suggested by the manufacturer (0.20-0.25) with an additional range on each side (0.15-0.225).

**Supplementary Figure 2.** Validation of FTIR Principal Component Analysis (PCA) clustering cut-off value. The most restrictive cut-off value that maximized the adjusted rand index (AR) was 0.146 for the clustering of the study dataset (*N*=150) of *S. pneumoniae* isolates considering Quellung reaction as the reference method.

**Supplementary Figure 3.** Validation of FTIR linear discriminant analysis (LDA) clustering cut-off value. The most restrictive cut-off value that maximized the adjusted rand index (AR) was 13.835 for the clustering of the study dataset (*N*=150) of *S. pneumoniae* isolates considering Quellung reaction as the reference method.


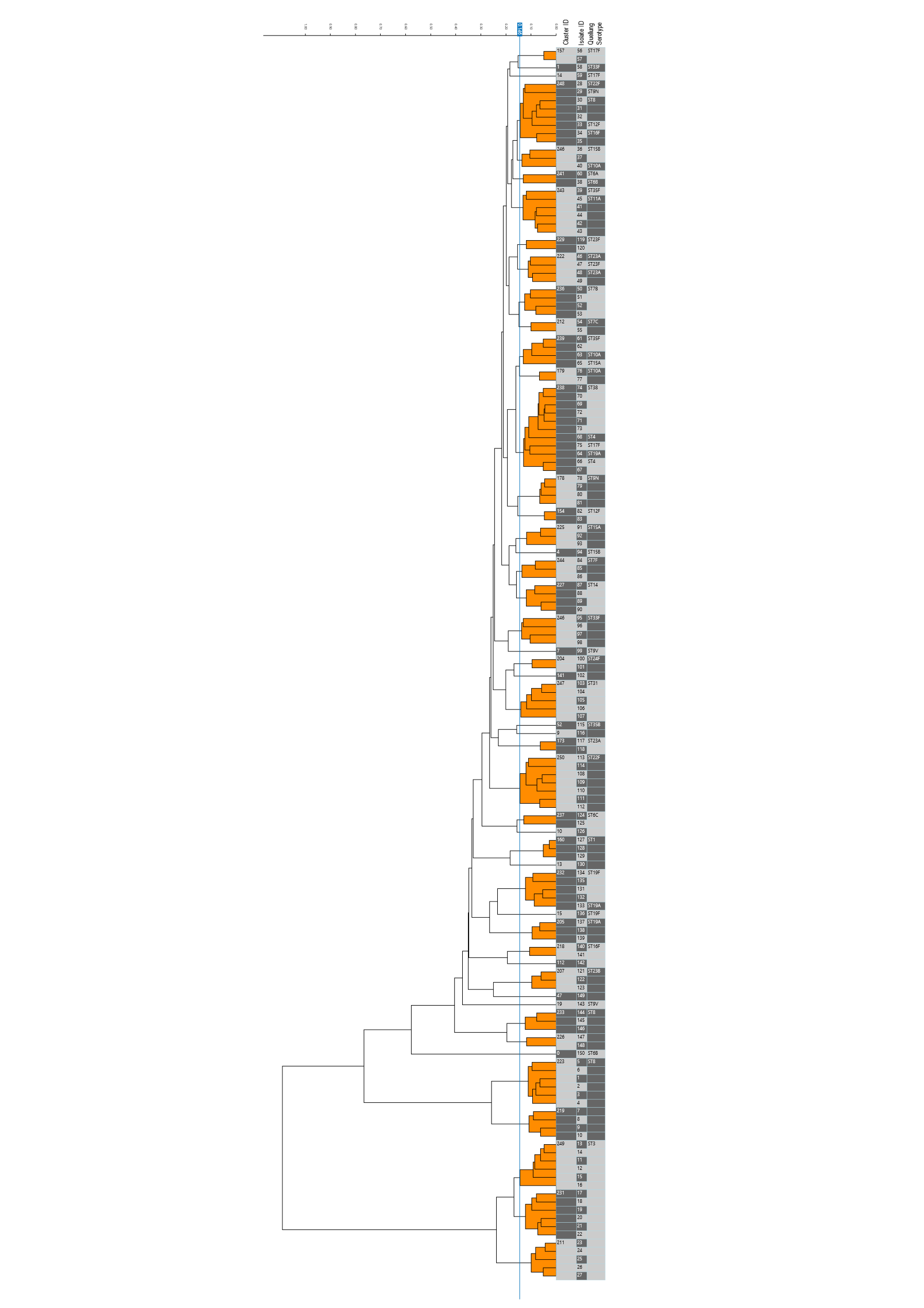


**Supplementary Figure 4.** Dendrogram visualizing the 150 tested pneumococcal strains representing 32 serotypes. The vertical dashed line indicates the cutoff value (0.146). The dendrogram was constructed using the Euclidean distance metric with Principal Component Analysis (PCA) as the dimensionality reduction method. The resulting PCA clusters, isolate IDs, and corresponding Quellung serotypes are indicated in each column.


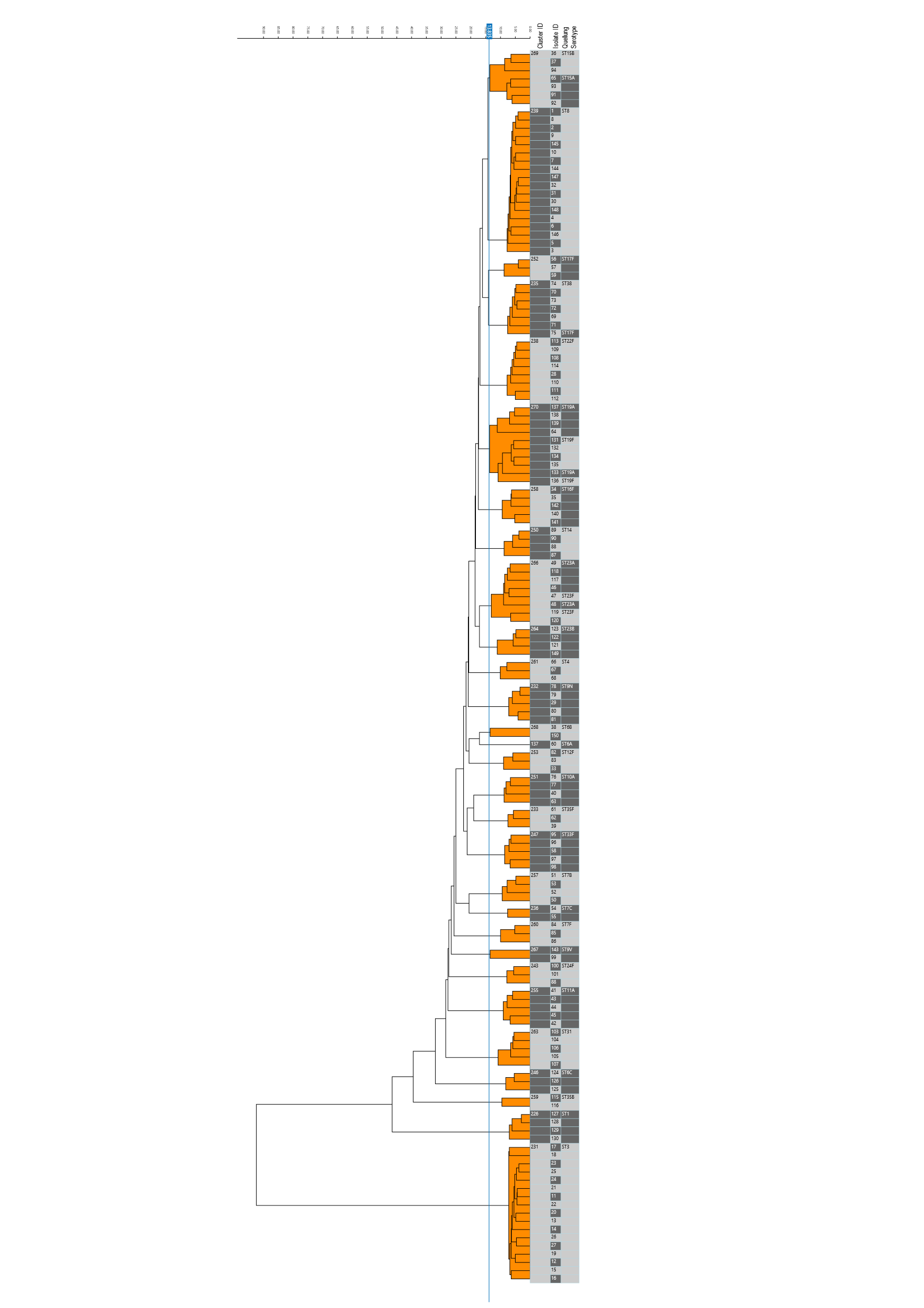


**Supplementary Figure 5.** Dendrogram visualizing the 150 tested pneumococcal strains representing 32 serotypes. The vertical dashed line indicates the cutoff value (13.835). The dendrogram was constructed using the Euclidean distance metric with linear discriminant analysis (LDA) as the dimensionality reduction method. The resulting LDA clusters, isolate IDs, and corresponding Quellung serotypes are indicated in each column.
